# Is it time to revisit the PaO_2_/FiO_2_ ratio to define the severity of oxygenation in ARDS?

**DOI:** 10.1186/s13613-021-00927-0

**Published:** 2021-09-22

**Authors:** Sunitha Palanidurai, Jason Phua, Yiong Huak Chan, Amartya Mukhopadhyay

**Affiliations:** 1 Intensive Care Unit, Alexandra Hospital, National University Health System, Singapore, Singapore; 2 FAST and Chronic Programmes, Alexandra Hospital, National University Health System, Singapore, Singapore; 3 Division of Respiratory & Critical Care Medicine, Department of Medicine, National University Hospital, National University Health System, Singapore, Singapore; 4 Yong Loo Lin School of Medicine, National University of Singapore, Singapore, Singapore; 5 Biostatistics Unit, Yong Loo Lin School of Medicine, National University of Singapore, Singapore, Singapore; 6 Medical Affairs, Alexandra Hospital, Singapore, Singapore

From the authors,

We appreciate Dr. El-Khatib et al.’s comments regarding our article on the “P/FP ratio” [1]. As we all agree [1–3], the ratio of the partial pressure of arterial oxygen (PaO2) to the fraction of inspired oxygen (FiO2), or P/F ratio, can be significantly improved for better reflection of acute respiratory distress syndrome (ARDS) severity by incorporating a wide range of either applied or measured pressures available in an intubated patient.

The oxygenation factor (OF) (PaO2/[FiO2*MAP]) proposed and studied by EL Khatib et al. is a reciprocal of the oxygenation index (OI) ([FiO2*MAP]/PaO2) which is often used in paediatric patients. Both indices incorporate mean airway pressure (MAP) into the P/F ratio. While we agree that for the same P/F ratio, a patient with higher MAP may have more severe ARDS than a patient with lower MAP, the use of MAP has several limitations. First, it is a very non-specific variable which is dependent on multiple factors, including tidal volume, inspiratory time, flow, respiratory rate, peak inspiratory pressure, and positive end-expiratory pressure (PEEP). Second, the impact of PEEP on PaO2 is much higher than any of these other variables. Third, when PEEP requirements go up, clinicians usually use various lung-protective strategies to limit alveolar pressure and its surrogate, plateau pressure (to less than 28 to 30 cmH2O)—this in turn limits the rise of MAP. Furthermore, in our dataset of 7 randomized controlled trials using receiver operating characteristics (ROC) curves, we found significantly better predictive validity for hospital mortality with the P/FP ratio compared to the OF or OI and the P/F ratio for different thresholds of PEEP > than 5 cmH2O, as measured by areas under the curves (AUC) (Fig. 1).

**Fig. 1:**
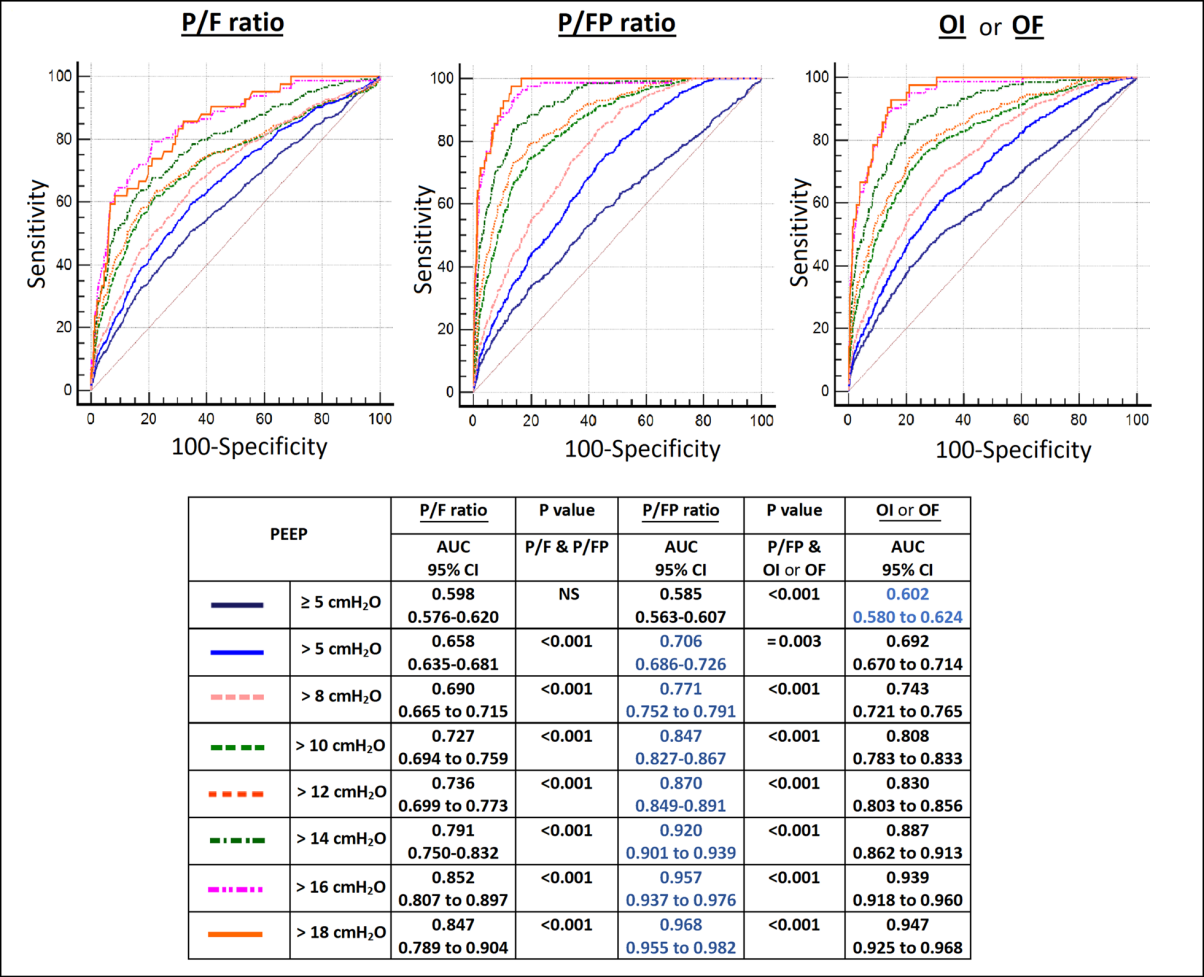
Receiver operating curves for hospital mortality at different PEEP thresholds for 3191 patients from our study [1], after excluding those with missing mean airway pressure values. Abbreviations: P/F, ratio of the partial pressure of arterial oxygen (PaO2) to the fraction of inspired oxygen (FiO2); P/FP = (PaO2*10)/(FiO2*PEEP); OI, oxygenation index = (FiO2*MAP)/PaO2; OF, oxygenation factor = (PaO2/(FiO2*MAP); PEEP, positive end-expiratory pressure; AUC, area under the curve; CI confidence interval; NS, not significant

We had provided several justifications in our paper for choosing the correction factor of 10 with our P/FP ratio. These include previous suggestions to use an applied PEEP of ≥ 10 cmH2O as an initial standardized ventilator setting for ARDS, and our own analysis of a regression line of PEEP versus P/F ratio in our dataset which found a clear intersection of a PEEP of 10 cmH2O and a P/F ratio of 150 mmHg, a value midway between 0 and 300 mmHg. The correction factor of 10 brings the values of the P/FP ratio to a range similar to that of the P/F ratio, which clinicians are already familiar with. For example, for a patient with a P/F ratio of 150 mmHg and a PEEP of 10 cmH2O, a correction factor of 10 would result in a P/FP ratio of (150/10)*10 = 150 mmHg/cmH2O. A correction factor of 5 would result in a P/FP ratio of (150/10)*5 = 75 mmHg/cmH2O, thus potentially giving clinicians a false impression of greater severity than is actually the case. A correction factor of 15 would result in a P/FP ratio of (150/10)*15 = 225 mmHg/cmH2O, thus potentially giving clinicians a false impression of milder severity than is actually the case. On the other hand, for a patient with a P/F ratio that is spuriously low at 150 mmHg as a result of an insufficient PEEP of 5 cmH2O, a correction factor of 10 would result in a P/FP ratio of (150/5)*10 = 300 mmHg/cmH2O, thus giving a better picture of the actual severity (or rather, lack thereof). In sum, the correction factor of 10 can be applied to any level of PEEP.

There is a wide variation in the practice of choosing PEEP globally. Although the concept of personalized PEEP settings continues to generate much interest, our study shows that the simple multifactorial P/FP ratio is significantly better able to predict mortality for ARDS than other related indices and ratios. As our study and El-Khatib et al.'s demonstrate, the P/F ratio needs to be revisited for better classification and prognostication of ARDS.

## Data Availability

ARDS network trials mentioned in our article [1].
